# Safety, pharmacokinetics, and pharmacodynamics of BMS-986142, a novel reversible BTK inhibitor, in healthy participants

**DOI:** 10.1007/s00228-017-2226-2

**Published:** 2017-03-06

**Authors:** Sun Ku Lee, Jun Xing, Ian M. Catlett, Robert Adamczyk, Amber Griffies, Ang Liu, Bindu Murthy, Miroslawa Nowak

**Affiliations:** Bristol-Myers Squibb, 3551 Lawrenceville Rd, Princeton, NJ 08540 USA

**Keywords:** BMS-986142, Drug–drug interaction, Methotrexate, Pharmacokinetics, Pharmacodynamics, Reversible Bruton’s tyrosine kinase inhibitors

## Abstract

**Purpose:**

BMS-986142 is an oral, small-molecule reversible inhibitor of Bruton’s tyrosine kinase. The main objectives of our phase I studies were to characterize the safety and tolerability, pharmacokinetics, and pharmacodynamics of BMS-986142 in healthy participants, and to investigate the potential for the effect of BMS-986142 on the PK of methotrexate (MTX) in combination.

**Methods:**

In a combined single ascending dose and multiple ascending dose study, the safety, pharmacokinetics, and pharmacodynamics of BMS-986142 were assessed in healthy non-Japanese participants following administration of a single dose (5–900 mg) or multiple doses (25–350 mg, once daily for 14 days). In a drug–drug interaction study, the effect of BMS-986142 (350 mg, once daily for 5 days) on the single-dose pharmacokinetics of MTX (7.5 mg) was assessed in healthy participants.

**Results:**

BMS-986142 was generally well tolerated, alone and in combination with MTX. BMS-986142 was rapidly absorbed with peak concentrations occurring within 2 h, and was eliminated with a mean half-life ranging from 7 to 11 h. Exposure of BMS-986142 appeared dose proportional within the dose ranges tested. A dose- and concentration-dependent inhibition of CD69 expression was observed following administration of BMS-986142. BMS-986142 did not affect the pharmacokinetics of MTX.

**Conclusions:**

BMS-986142 was well tolerated at the doses tested, had pharmacokinetic and pharmacodynamic profiles which support once-daily dosing, and can be coadministered with MTX without the pharmacokinetic interaction of BMS-986142 on MTX.

**Electronic supplementary material:**

The online version of this article (doi:10.1007/s00228-017-2226-2) contains supplementary material, which is available to authorized users.

## Introduction

Rheumatoid arthritis (RA) is a chronic, systemic autoimmune disease affecting 0.5–1.0% of the population in industrialized countries [1]. Synovial inflammation leads to articular pain, swelling, and structural destruction that contribute to reduced quality of life [2]. Treatment includes monotherapy or combination therapy with classical disease-modifying anti-rheumatic drugs (DMARDs) and biologic agents, including tumor necrosis factor alpha (TNFα) blockers, anti-cytokine therapies, B cell depleting agents, and costimulation molecule modulation agents [3–7]. Currently, methotrexate (MTX) remains the cornerstone of RA treatment [6, 7]. Despite recent progress in RA therapy, some patients either do not reach the treatment targets of low disease activity or remission, or experience drug toxicities, or both [8]. Given the complexity, chronicity, and progressive nature of RA, new safe and efficacious therapies with novel mechanisms of action that complement existing treatments are needed.

Bruton’s tyrosine kinase (BTK) is a member of the Tec family of nonreceptor tyrosine kinases and is expressed in all hematopoietic cells except T cells and terminally differentiated plasma cells. BTK inhibition is expected to impact mechanisms involving B cell- and non B cell-mediated autoimmunity such as RA and lupus via B cell receptor, Fc receptor, and RANK receptor signaling [9–12].

BMS-986142 is an oral, small-molecule reversible inhibitor of the kinase activity of BTK that, unlike rituximab—a B cell-depleting, anti-CD20 antibody [13]—is expected to inhibit antigen-dependent B cell signaling without depleting B cells. Added benefit against disease endpoints in the collagen-induced arthritis model was evident when BMS-986142 was combined with other agents representing the standard of care (e.g., MTX), suggesting BMS-986142 may be an effective therapy for RA. Overall, it appears that BTK is an attractive novel therapeutic target for RA and other autoimmune diseases.

Two phase I studies were conducted to characterize the safety and pharmacokinetic (PK)/pharmacodynamic (PD) profile of BMS-986142 in healthy participants, and its drug–drug interaction (DDI) potential (reported only for MTX), in order to inform future clinical development. Overall study design is described in “Methods.” The focus of this report is to evaluate the safety, tolerability, PK, and PD of BMS-986142 after single ascending dose (SAD) and multiple ascending dose (MAD) administration in healthy participants in study 1, and to evaluate the effect of BMS-986142 on the single-dose PK of MTX (the standard of care in RA patients) in healthy participants in study 2. The reason for prioritizing this data was the following: the SAD/MAD data provides a critical evaluation of the PK, PD and safety profile of the BTK inhibitor. Secondly, the MTX-DDI results are relevant to disclose considering the target population. The remaining portion of studies 1 and 2 will be presented separately from this report.

## Methods

### Overall study design of two phase I studies

Study 1 was a first-in-human study including SAD and MAD in non-Japanese participants, MAD in Japanese participants, and relative bioavailability with food effect in non-Japanese participants. Study 2 was a DDI study evaluating the effect of BMS-986142 on the PK of MTX and cocktail probes for multiple metabolic enzymes and transporters. Herein, we present the detailed study design for the portion of SAD and MAD in Study 1, a phase I, randomized, double-blind, placebo-controlled study in healthy participants (NCT02257151; data on file), and for the portion of MTX-DDI in study 2, a phase I, open-label, single-sequence DDI study in healthy participants (NCT02456844; data on file). Below, we present the detailed methods presented in this report.

### Study design of SAD and MAD in study 1

Eligible participants were confined to the clinical facility (WCCT Global, LLC, Cypress, CA) the day before dosing (day −1) and randomized to receive SAD or MAD treatment under fasting conditions for 10 h. In the SAD group, eight healthy non-Japanese participants were assigned to each of up to six sequential dose panels (single oral doses of 5, 15, 50, 100, 300, and 900 mg BMS-986142) or placebo. Within each dose panel, participants were randomized in a 3:1 ratio to receive BMS-986142 (*n* = 6) or placebo (*n* = 2). In the MAD group, eight healthy non-Japanese participants were assigned to each of four sequential dose panels (daily oral doses of 25, 75, 200, and 350 mg BMS-986142) or placebo. Within each dose panel, participants were randomized in a 3:1 ratio to receive once-daily (QD) doses of BMS-986142 or placebo from day 1 to day 14 (Online Resource 1). Escalation to the next dose level in SAD and MAD was conducted after safety assessment had been completed and the treatment was considered safe and well tolerated. Additionally, dose levels in MAD were selected not to exceed the steady-state exposure of BMS-986142 (AUC: 22,920 ng h/mL) at NOAEL in rats obtained from a 1-month GLP toxicology study.

Eligible participants were healthy males or females (not of childbearing potential) ages ≥18 to ≤55 years, with a body mass index of ≥18.0 to ≤32.0 kg/m^2^, whose health status was determined by medical history, surgical history, physical examination, vital signs, electrocardiograms (ECGs), chest X-ray, and clinical laboratory assessments. Exclusion criteria included administration or plans for administration of live vaccine to participants or their household contacts 12 weeks before or 30 days after the last dose of the study drug; treatment for active tuberculosis within the previous 3 years; history of herpes zoster; acute or chronic bacterial infection; upper respiratory infection; known or suspected infection with human immunodeficiency virus-1 or virus-2, or hepatitis B or C viruses; suspected autoimmune disorder; abnormal routine clinical laboratory values, thyroid function test, or electrolytes; and chronic use of steroids.

### Study design of MTX-DDI in study 2

This portion of the study was designed to assess the effect of BMS-986142 on the PK of MTX since MTX is used as a background therapy in RA patients and all patients will receive MTX when the phase 2 study of BMS-986142 is conducted. Eligible participants were confined to the clinical facility for the duration of treatment (15 days). Healthy participants were administered a single oral dose of 7.5 mg MTX on day 1. On day 2, participants received leucovorin (a single oral dose of 15 mg) as a preventive measure for MTX toxicity. After a washout period (days 2–5), BMS-986142 was administered on days 6–10 in 350-mg QD doses. A concomitant dose of MTX (7.5 mg) was administered on day 8, followed by leucovorin (15 mg) on day 9 (Online Resource 2). BMS-986142 was administered with 240 mL of water, and a minimum amount of additional water was permitted to allow dosing of all medications. Only healthy male participants (ages ≥18 to ≤50 years) were eligible for the MTX-DDI assessment in study 2. Other key inclusion and exclusion criteria were identical to those in study 1.

### Safety analyses

Participants were monitored for adverse events (AEs) throughout the study. Clinical laboratory tests, vital sign measurements, 12-lead ECGs, and physical examinations were performed at selected times throughout the dosing interval. Safety data was recorded in the participants’ medical records, and assessments were performed for emergent AEs or serious AEs (SAEs). For all AEs, causal relationship to the study drug was determined by an investigator. QT signals obtained from 12-lead ECGs were corrected by heart rate using Fridericia’s formula (QTcF). Baseline-corrected QTcF (∆QTcF) were evaluated to determine QT elongation.

### Pharmacokinetic assessments

Blood samples for the measurement of BMS-986142 were collected up to 168 h after dosing of BMS-986142 on day 1 for participants in SAD. In MAD, blood samples were collected up to 24 h on day 1 and up to 120 h on day 14 (the last day of treatment). Blood samples for the measurement of MTX were collected up to 48 h after dosing of MTX on days 1 and 8.

Plasma samples were analyzed for BMS-986142 levels with a validated method using liquid chromatography tandem mass spectrometry (LC-MS/MS) detection. The analyte was extracted from 50 μL of plasma using a liquid–liquid extraction method. Methyl tert-butyl ether was used as the extraction solvent. The lower limit of quantification (LLOQ) was 1.00 ng/mL in plasma. Plasma samples were analyzed for methotrexate levels using LC-MS/MS detection. Analytes were isolated from 50 μL plasma through solid phase extraction using Waters Oasis MAX, 10-mg, 96-well SPE plates. The final extract was analyzed using LC-MS/MS detection. LLOQ was 1.00 ng/mL in plasma. Detailed methodology quantitating the concentration level of BMS-986142 and MTX is provided in Online Resource 10.

Individual subject pharmacokinetic parameters for BMS-986142 and MTX were derived by a non-compartmental method using validated PK software (WinNonLin version 6.3; Pharsight Corporation, Mountain View, CA, USA). The following PK parameters for BMS-986142 and MTX were derived from plasma concentration versus time data: maximum observed plasma concentration (C_max_); minimum observed plasma concentration (C_min_); time to maximum observed plasma concentration (T_max_); area under the plasma concentration-time curve from time zero to the time of the last quantifiable concentration (AUC_(0-T)_), from time zero extrapolated to infinite time (AUC_(inf)_), and in one dosing interval (AUC_(TAU)_); terminal plasma half-life (T_1/2_); and AUC accumulation index (AI_AUC). AI_AUC was calculated to divide AUC_(TAU)_ on day 14 by AUC_(TAU)_ on day 1.

### Pharmacodynamic assessments

To assess the functional effect of BMS-986142 on a relevant pathway in the targeted cells, whole blood was stimulated ex vivo with anti-IgD-dextran to activate signal transduction through the B cell antigen receptor. B cells, identified by CD20, were assessed for expression of CD69. Blood was incubated without addition of anti-IgD-dextran to serve as a negative control. Samples were incubated for 24 h at 37 °C, and then fixed and stained for CD20 and CD69. CD69 expression was quantified by standardized flow cytometry. Fluorescence intensity was measured and reported in units of mean equivalents of soluble fluorescein (MESF). Background CD69 level was measured from samples without the addition of anti-IgD-dextran and was subtracted from the stimulated value. The anti-IgD specific CD69 induction was used for analysis. The result for each sample was expressed as a percentage inhibition relative to the pre-dose sample.

### Statistical methods

All recorded AEs were listed and tabulated by system organ class, preferred term, and treatment. The number and percentage of participants with marked laboratory abnormalities were summarized.

Summary statistics were tabulated for all PK parameters by treatment and study days. Geometric mean (Gmean) and coefficient of variation (CV) are presented for C_max_, AUC_(0-T)_, AUC_(inf),_ AI_AUC, C_min_, and AUC_(TAU)_; arithmetic mean and standard deviation (SD) are presented for T_1/2_; and median and range are presented for T_max_. Dose proportionality was assessed using the previously described power model [14]. Briefly, the simple linear regression was applied to the natural log of the PK parameters on the natural log of dose using the following equation (Eq. 1).

1 $$ \mathrm{E}\left[ \log \left(\mathrm{PK}\ \mathrm{Parameter}\right)|\mathrm{Dose}\right]=\alpha +\beta \ast \log \left(\mathrm{Dose}\right). $$

A slope (β) equal to 1 would indicate perfect dose proportionality. For each PK parameter (C_max_, AUC_(0-T)_, AUC_(TAU)_, and AUC_(inf)_), point estimates and 90% CI of the slopes were calculated.

CD69 inhibition by BMS-986142 was tabulated by treatment and time, and corresponding changes from baseline were calculated and summarized. The inhibition of B cell receptor-mediated CD69 expression by BMS-986142 was descriptively summarized by treatment and study days. An exposure-response analysis was applied to characterize the relationship between BMS-986142 concentration and CD69 inhibition using a Bayesian Emax model. The Bayesian Emax model as described below (Eq. 2) was fit to the matched BMS-986142 plasma concentration and CD69 percent inhibition data from SAD and MAD panels, and posterior probabilities of hitting expected target inhibition was estimated. Bayesian analysis was selected to support the SAD and MAD study. Compared to the conventional approach, Bayesian approach provides flexibility to adjust dose levels and efficiently characterize PK and PD profile during the study process.

2 $$ I\mathrm{NH}=\mathrm{E}0+\frac{{\mathrm{Emax}\times \mathrm{Concentration}}^{\mathrm{Hill}}}{\mathrm{EC}{50}^{\mathrm{Hill}}+{\mathrm{Concentration}}^{\mathrm{Hill}}} $$

where INH = CD69 inhibition response, E0 = response at placebo, Emax = maximum response, and Hill = steepness parameter.

Statistical analyses were conducted to assess the effect of coadministration of BMS-986142 on the PK of MTX. For all treatment comparisons, a linear mixed-effect model was fitted to the log-transformed PK parameters C_max_, AUC_(0-T)_, and AUC_(inf)_. Point estimates and 90% CIs for differences on the log-scale were exponentiated to obtain estimates for geometric mean ratios (GMRs) and respective 90% CIs on the original scale. Sample size determination is based on consideration of the precision of the estimate of the GMRs of AUCs of MTX with and without BMS-986142. With 9 evaluable subjects, there will be an 80% probability that the 90% CI of AUC(INF) GMR will be within 89.9 to 111.2% of the point estimate. These precision estimates are based on an assumption that Cmax and AUC(INF) of MTX are log-normally distributed with intrasubject CV of 14.67 and 10.38%, respectively, as calculated from Namour et al. [15].

SAS® version 9.2 or greater (SAS Institute Inc., Cary, NC) was used for statistical analyses, tabulations and graphical presentations. R 3.0.1 or greater was used for the Bayesian Emax model.

## Results

### Patient characteristics and disposition

A total of 80 participants were enrolled in study 1: 48 patients in the SAD group (6 participants in each BMS-986142 dose group, 12 in the placebo group), and 32 in the MAD group (6 participants in each BMS-986142 group, 8 in the placebo group). A total of 12 participants were enrolled in study 2, in which the DDI potential of single 7.5-mg doses of MTX alone and in combination with BMS-986142 (350 mg QD) was evaluated (Online Resource 3).

### Safety and tolerability

In the study 1 SAD group, 22 AEs were reported in 8 participants (22.2%) treated with BMS-986142, and 6 AEs were reported by 2 participants (16.7%) treated with the placebo (Table 1, Online Resource 4). Two of these AEs were of grade 2 severity (syncope, placebo group; headache, 50-mg BMS-986142 group); all remaining AEs were of grade 1 severity. No clinical symptoms were associated with laboratory abnormalities.

**Table 1:** Overall safety summary

	Study 1				Study 2
	SAD		MAD		
Adverse event (AE), *n* (%)	Placebo *n* = 12	BMS-986142 *n* = 36	Placebo *n* = 8	BMS-986142 *n* = 24	BMS-986142 *n* = 12
Subjects with AEs	2 (16.7)	8 (22.2)	4 (50)	8 (33.3)	4 (33.3)
Dizziness	0	1 (2.8)	0	0	2 (16.7)
Headache/tension headache	1 (8.3)	5 (13.9)	1 (12.5)	1 (4.2)	1 (8.3)
Nausea	0	1 (2.8)	0	0	2 (16.7)
Diarrhea	0	1 (2.8)	0	1 (4.2)	0
Cough	0	1 (2.8)	1 (12.5)	1 (4.2)	0
Upper respiratory tract infection	0	2 (5.6)	0	1 (4.2)	0
Thermal burns	0	1 (2.8)	0	1 (4.2)	0

*MAD* multiple ascending dose, *SAD* single ascending dose

In the study 1 MAD group, 13 AEs were reported in 8 participants (33.3%) treated with BMS-986142, and 5 AEs were reported in 4 participants (50%) treated with the placebo (Table 1, Online Resource 4). One subject who received multiple doses of BMS-986142 (75 mg, QD) had a grade 3 SAE of a brief psychotic disorder occurring 26 days after the last administration of the study drug. This event occurred shortly after an esophagogastroduodenoscopy procedure that the subject underwent as a participant of another clinical study. The event was considered not related to the study drug by the investigator. One subject had a grade 3 AE of increased blood creatine phosphokinase that was considered unrelated to the study drug by the investigator. Two AEs were of grade 2 severity (syncope, placebo group; rash, 350-mg BMS-986142 group), while all remaining AEs were of grade 1 severity in the MAD group.

Alanine aminotransferase (ALT) elevation was identified during the study as an event of special interest. Modest elevation of ALT (<3× upper limit of normal [ULN]) was noted and the frequency of marked ALT elevation was low. The highest measured level of ALT was 177 units per liter (U/L) (between ALT ≥3× and <5× ULN, where ULN = 55 U/L) and occurred in the placebo group the SAD portion of the study. The same subject had an AST elevation (72 U/L) that was approximately 1.5× ULN (ULN = 50 U/L). No clinically relevant changes of QTcF from baseline were observed in SAD and MAD (Online Resources 5 and 6). No participants showed greater than 30 ms increase from baseline in the maximum post-dose QTcF.

In study 2, the DDI study with MTX, four participants experienced 12 AEs of grade 1 severity that did not require any treatment or dose adjustment. Three AEs were reported in 1 subject who received single-dose MTX on day 1, 2 AEs were reported in 2 participants who received BMS-986142 administration on days 6 and 7, and 9 AEs were reported in 4 participants who received concomitant MTX and BMS-986142 (days 8–10). Among reported AEs, the most common (≥2 episodes) were dizziness, headache, and nausea. Two participants experienced AEs related to the study drug. These were dizziness and nausea reported in both participants, occurring after administration of MTX with BMS-986142. All other drug-related AEs after coadministration of MTX with BMS-986142 were reported in 1 subject each. There were no drug-related AEs after administration of MTX alone or BMS-986142 alone (Table 1, Online Resource 7 **)**. Overall, BMS-986142 and MTX were well tolerated when administered alone or in combination.

### Pharmacokinetics

**Fig. 1:**
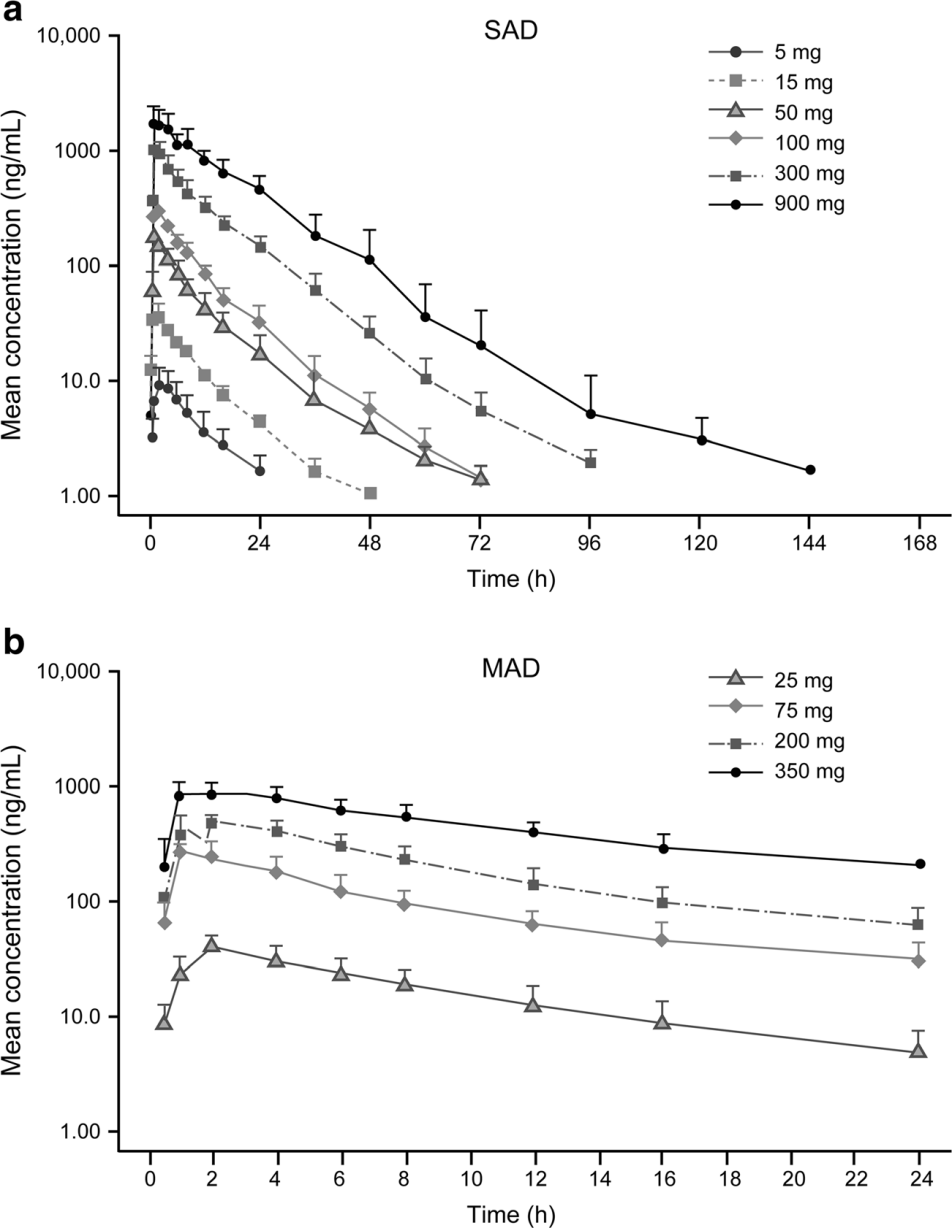
Mean concentration vs. time profiles of BMS-986142 after **a** SAD and **b** MAD administration. Lower limit of quantification (LLOQ) = 1.00 ng/mL. *MAD* multiple ascending dose, *SAD* single ascending dose

Mean concentration versus time profile after a single-dose administration is presented in Fig. 1a. Following single-dose administration of BMS-986142 over the 5–900 mg dose range, BMS-986142 was rapidly absorbed (median T_max_ up to 2 h). Mean T_1/2_ ranged 7 to 11 h (Table 2). Increases in C_max_ and AUC_(inf)_ after a single-dose administration appears to be approximately dose proportional from 5 to 900 mg of BMS-986142 as the slope of the regression line was close to 1 and the corresponding confidence interval was entirely contained within 0.80 to 1.25 (Online Resource 8, Online Resource 9).

**Table 2:** Pharmacokinetic parameters for BMS-986142 after (a) single-dose administration and (b) at steady state after multiple-dose administration

a. SAD, day 1						
BMS-986142 treatment dose	C_max_ (ng/mL) geometric mean (%CV)	T_max_ (h) median (min, max)	AUC_(0-T)_ (ng h/mL) geometric mean (%CV)	AUC_(inf)_ (ng h/mL) geometric mean (%CV)	T_1/2_ (h) mean (SD)	
5 mg, *n* = 6	9.03 (37)	2.03 (1.00, 4.00)	83.1 (51)	102 (52)^a^	6.87 (3.35)^a^	
15 mg, *n* = 6	36.9 (21)	2.00 (1.00, 2.00)	369 (12)	388 (10)	8.02 (1.37)	
50 mg, *n* = 6	185 (38)	1.00 (1.00, 2.05)	1561 (31)	1586 (31)	9.70 (1.65)	
100 mg, *n* = 6	311 (17)	1.50 (1.00, 2.00)	2999 (14)	3019 (14)	10.3 (1.03)	
300 mg, *n* = 6	1081 (25)	1.02 (1.00, 2.00)	11,347 (21)	11,390 (21)	10.6 (1.05)	
900 mg, *n* = 6	1878 (31)	2.00 (1.00, 4.00)	28,026 (32)	28,063 (31)	10.7 (2.01)	
b. MAD, day 14						
BMS-986142 treatment dose	C_max_ (ng/mL) geometric mean (%CV)	T_max_ (h) median (min, max)	C_min_ (ng/mL) geometric mean (%CV)	AUC_(TAU)_ geometric mean (%CV)	AI_AUC geometric mean (%CV)	T_1/2_ (h) mean (SD)
25 mg, *n* = 6	56.0 (25)	2.00 (2.00, 4.00)	7.85 (42)	539 (34)	1.57 (22)	10.9 (1.8)
75 mg, *n* = 6	281 (34)	2.00 (1.00, 2.00)	27.9 (77)	2471 (44)	1.23 (31)	10.7 (1.6)
200 mg, *n* = 6	592 (37)	2.00 (1.00, 2.00)	67.6 (63)	5527 (41)	1.23 (40)	11.0 (4.6)
350 mg, *n* = 6	1024 (14)	1.50 (1.00, 2.00)	166 (41)	10,715 (26)	0.984 (32)	10.2 (0.9)

*AI_AUC* AUC accumulation index, *AUC*_*(0-T)*_ area under the plasma concentration-time curve from time zero to the time of the last quantifiable concentration, *AUC*_*(inf)*_ area under the plasma concentration-time curve from time zero extrapolated to infinite time, *AUC*_*(TAU)*_ area under the plasma concentration-time curve in one dosing interval, *C*_*max*_ maximum observed plasma concentration, *C*_*min*_ minimum observed plasma concentration, *MAD* multiple ascending dose, *SAD* single ascending dose, *T*_*1/2*_ terminal plasma half-life, *SD* standard deviation, *T*_*max*_ time to maximum observed plasma concentration

^a^ *n* = 5

Mean concentration versus time profile at steady state following multiple doses is presented in Fig. 1b. After multiple-dose administration, steady-state PK behavior was consistent with that following a single-dose administration. (Table 2). Dose-proportional increases in C_max_ and AUC_(TAU)_ at steady state were observed from 25 to 350 mg as the slope of the regression line was close to 1 and the corresponding confidence interval included 1 (Online Resource 8, Online Resource 9). No changes in T_max_ and T_1/2_ were observed in comparison to the SAD part of the study. The accumulation index (AI) for AUC indicated a minimal to modest accumulation in MAD.

**Fig. 2:**
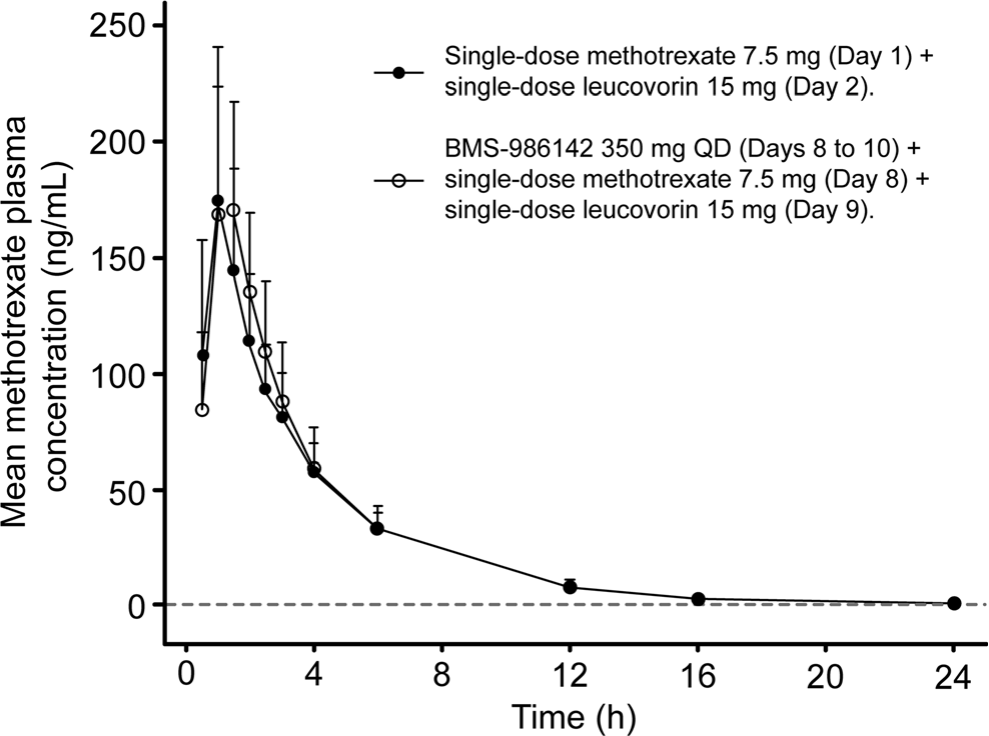
Plasma methotrexate (MTX) concentration vs. time curves for single-dose administration and coadministration with BMS-986142. *QD* once daily; LLOQ = 1.00 ng/mL

MTX exposures were comparable when administered alone or with BMS-986142. The coadministration of BMS-986142 did not appear to affect the Cmax and AUC(inf) of MTX as GMRs approximated 1 and the 90% CIs of the adjusted GMRs were entirely contained within 0.80 to 1.25 (Table 3). Plasma MTX concentration versus time curves for single-dose administration and coadministration with BMS-986142 were superimposable (Fig. 2).

**Table 3:** Adjusted geometric mean ratios for C_max_ and AUC of methotrexate administered as a single dose and in combination with BMS-986142

PK parameter	Treatment	Geometric mean (adjusted)	90% CI
C_max_ (ng/mL)	D	178	(154, 205)
	F	186	(166, 209)
	F vs D	1.046	(0.910, 1.202)
AUC_(0-T)_ (ng h/mL)	D	613	(550, 683)
	F	638	(568, 717)
	F vs D	1.041	(0.950, 1.139)
AUC_(inf)_ (ng h/mL)	D	625	(563, 695)
	F	654	(583, 733)
	F vs D	1.045	(0.954, 1.145)

*AUC*_*(0-T)*_ area under the plasma concentration-time curve from time zero to the time of the last quantifiable concentration, *AUC*_*(inf)*_ area under the plasma concentration-time curve from time zero extrapolated to infinite time, *CI* confidence interval, *C*_*max*_ maximum observed plasma concentration, *PK* pharmacokinetic. Treatments: *D* single-dose methotrexate 7.5 mg (day 1) + single-dose leucovorin 15 mg (day 2), *F* BMS-986142 350 mg QD (days 8 to 10) + single-dose methotrexate 7.5 mg (day 8) + single-dose leucovorin 15 mg (day 9)

### Pharmacodynamics

**Fig. 3:**
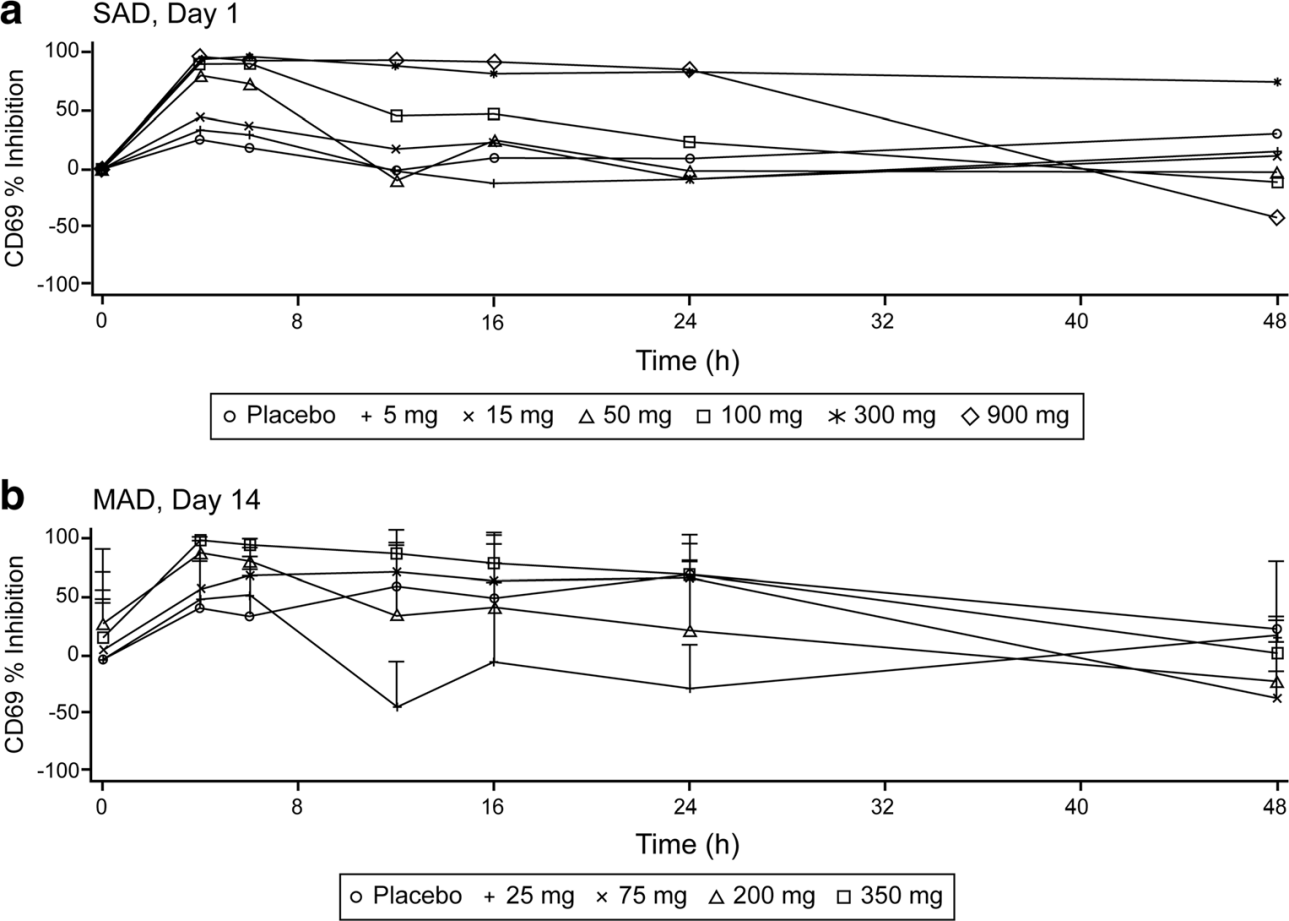
Change in CD69 expression vs. time after **a** single ascending dose (SAD) and **b** multiple ascending dose (MAD) administration

**Fig. 4:**
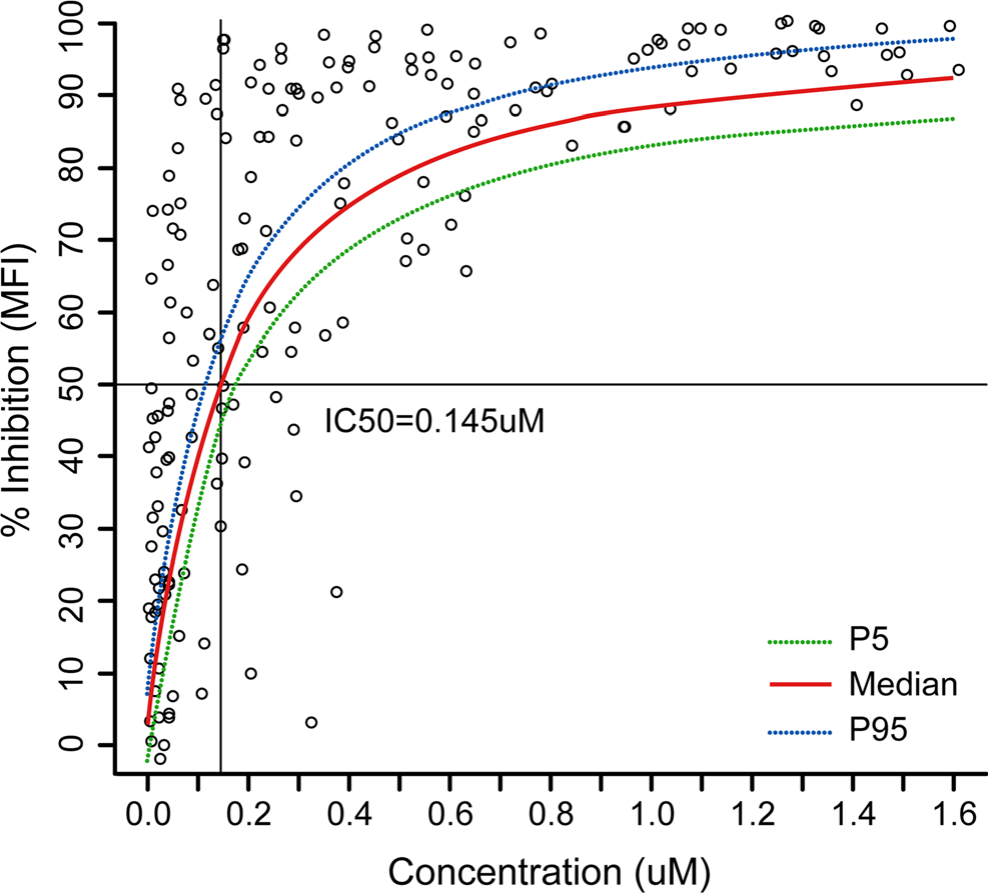
BMS-986142 plasma concentration and CD69% inhibition relationship curve. Data included participants with matching concentration of BMS-986142 and CD69 inhibition. Placebo participants were excluded from the analysis. Data excluded values of CD69% inhibition >100 or ≤−100. For illustration purposes, *Y-axis* only presented from 0 to 100%. IC50 = BMS-986142 concentration to inhibit the 50% of CD69 expression

Dose- and concentration-dependent inhibition of ex vivo anti-IgD-dextran induced CD69 expression were observed following single- or multiple-dose administration of BMS-986142. Mean maximal inhibition was close to 100% at dose levels greater than 100 mg, and sustained inhibition over a dosing interval was observed at steady state following multiple dosing at the 350-mg dose level (Fig. 3). Based on the relationship between concentration of BMS-986142 and corresponding inhibition of CD69 expression, the concentration to inhibit 50% of CD69 expression was 0.145 μM [83 ng/mL] (Fig. 4). Plasma concentration above 0.145 μM [83 ng/mL] for the entire duration at steady state was achieved at doses at or above 350 mg QD.

## Discussion

In the current study, BMS-986142 was generally well tolerated and exhibited a dose- and concentration-dependent inhibition of CD69 expression. Overall, the PK and PD profiles of BMS-986142 following single or multiple dosing supported once-daily dosing for future clinical studies. BMS-986142 did not affect the PK of MTX, and coadministration of BMS-986142 and MTX was generally well tolerated.

In healthy persons, B cells, and BTK, are not active. Thus, measurement of in vivo inhibition is problematic. To work around this issue, B cell activity was stimulated in vitro and CD69 expression was evaluated. CD69 expression can be induced downstream of the B cell receptor; thus, suppression of CD69 expression reflects inhibition of B cell activation. Since BTK is required for B cell receptor-mediated activation, it can serve as a surrogate marker for target (BTK) engagement [16]. BMS-986142 produced a dose- and concentration-dependent decrease in CD69 expression, indicating an ex vivo target engagement. Based on PK/PD analysis in SAD and MAD, the derived IC_50_ was 0.145 μM. This value was similar with that derived from in vitro evaluation (0.090 μM) [17]. It appears that a 350-mg QD dosing regimen is able to maintain plasma concentrations above the IC50 of CD69 inhibition over the entire dosing interval at steady state.

Inhibition of BTK-dependent processes such as B cell receptor-mediated B cell functions, IgG-containing immune complex signaling through Fcγ receptors in monocytic cells, and RANK-dependent osteoclastogenesis is expected to provide benefit in the treatment of autoimmune disorders such as RA, where these pathways are involved in disease pathogenesis [9, 10]. Dose-dependent efficacy of other BTK inhibitors such as PCI-32765 and GDC-0834 has been demonstrated in animal models of arthritis [18, 19]. Further, the BTK inhibitor RN486, when administered alone or in combination with MTX, was efficacious in reducing joint and systemic inflammation in a rat adjuvant-induced arthritis model [20]. Using a collagen-induced arthritis model, newer understanding of BTK function with inhibitors (CGI1746) for B cell or myeloid cell-driven diseases is generating compelling evidence and rationale for targeting BTK in RA [21]. Exploration of BTK inhibitors to treat RA is ongoing in nonclinical and clinical studies [22, 23].

There was a single SAE (psychotic event requiring hospitalization) reported during the study. The SAE occurred in the MAD 75-mg group and was considered unrelated to the study drug. It occurred 26 days after the last dose of the study drug and approximately 1 day following a gastroscopy procedure that the subject underwent as a participant of another clinical study. No dose-limiting AEs or clinically significant laboratory abnormalities were observed. No participants had an ALT >3× ULN and a total bilirubin >2× ULN on the same date. There were no apparent trends in vital signs or laboratory values following single- and multiple-dose administration of BMS-986142 and no clinically relevant changes in individual ECG intervals or changes from baseline were noted. There appeared to be a relationship between change from baseline in QTcF interval and BMS-986142 plasma concentration following single-dose administration of BMS-986142, but there was no apparent relationship following multiple dosing of BMS-986142. Similar trends were noted following model-based assessment [24]. These results will be explored further via a formal, thorough QT study.

The observed PK profile of BMS-986142 supports QD dosing in future clinical studies. Dose-proportional increases in C_max_ and AUCs were demonstrated following single- or multiple-dose administration, and multiple QD dosing of BMS-986142 led to modest accumulation at therapeutically relevant dose levels. Mean T_1/2_ was up to 11 h at therapeutic doses. At low dose levels, a shorter T_1/2_ (∼7 h) was observed. It is likely that terminal phase may not be fully characterized at these dose ranges since concentration levels at terminal phase may be below the LLOQ. Considering the observed T_1/2_, the modest accumulation of BMS-986142 was predicted following a QD dosing. The observed range of accumulation is consistent with the prediction. However, it should be noted that AI values appear to decrease with increasing dose levels. There may be dynamic changes such as the inhibition and/or induction of metabolic enzymes associated with the elimination of BMS-986142. These changes may be dose-dependent. These findings will be explored further in the future studies. It appears that the increases in Cmax were approximately dose proportional but showed the wide fluctuation across the dose range in SAD. Therefore, the dose-proportional increase in Cmax is not conclusive in SAD. MTX is the standard of care for treatment of RA; therefore, RA patients commonly receive it as a background therapy. Hence, confirming whether BMS-986142 affects the PK of MTX was important before conducting a clinical study in RA patients. Results from our current study demonstrated that BMS-986142 did not affect the PK of MTX, and coadministration with MTX was well tolerated. These results suggest that BMS-986142 can be administered safely to RA patients receiving MTX as a background therapy without restrictions or dosage adjustments.

## Conclusion

In summary, BMS-986142 was well tolerated and also produced sustained inhibition of CD69 expression. The PK profile of BMS-986142 was linear, supporting once-daily dosing, and no PK interaction with MTX was observed. Dose- and concentration-dependent inhibition of CD69 expression, representing target engagement, was observed. Collectively, these results—combined with reports from other BTK inhibitors—support the further development of BMS-986142 for the treatment of RA and other inflammatory diseases.

## Electronic supplementary material

Online Resource 1 Overall Study 1 design. Demarcation represents single and multiple ascending dose study (SAD and MAD). *D* study discharge, *MAD* multiple ascending dose, *R* randomization, *S* screening, *SAD* single ascending dose, *SDD* spray dried dispersion, *W* washout (GIF 74 kb)

High Resolution (TIFF 544 kb)

Online Resource 2 Study 2 design. Demarcation represents methotrexate drug-drug interaction study (MTX DDI). *DDI* drug–drug interaction, *MTX* methotrexate, *W* washout (GIF 65 kb)

High Resolution (TIFF 510 kb)

Online Resource 3 (DOCX 41 kb)

Online Resource 4 (DOCX 50 kb)

Online Resource 5 (DOCX 40 kb)

Online Resource 6 (DOCX 40 kb)

Online Resource 7 (DOCX 41 kb)

Online Resource 8 Dose proportionality (fitted regression line) of C_max_ and AUC with a) single ascending dose and b) multiple ascending dose administration (JPEG 141 kb)

High Resolution (TIFF 427 kb)

Online Resource 9 (DOCX 41 kb)

Online Resource 10 Analytical methods for the determination of BMS-986142 or MTX in the human plasma (DOCX 50 kb)
