# Supplementary figures and images for: Safety, pharmacokinetics, and pharmacodynamics of BMS-986142, a novel reversible BTK inhibitor, in healthy participants
Source: Eur J Clin Pharmacol. 2017 Mar 6;73(6):689–98. doi: 10.1007/s00228-017-2226-2 (PMC5423977; doi:10.1007/s00228-017-2226-2)

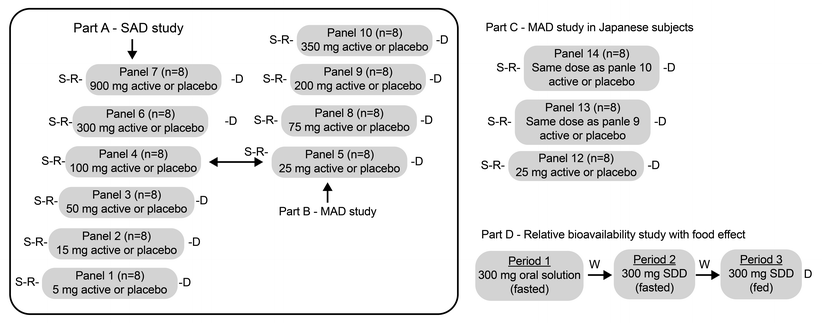

Supplement: Supplementary file 1 — Overall Study 1 design. Demarcation represents single and multiple ascending dose study (SAD and MAD). D study discharge, MAD multiple ascending dose, R randomization, S screening, SAD single ascending dose, SDD spray dried dispersion, W washout (GIF 74 kb) [file 228_2017_2226_Fig5_ESM.gif]

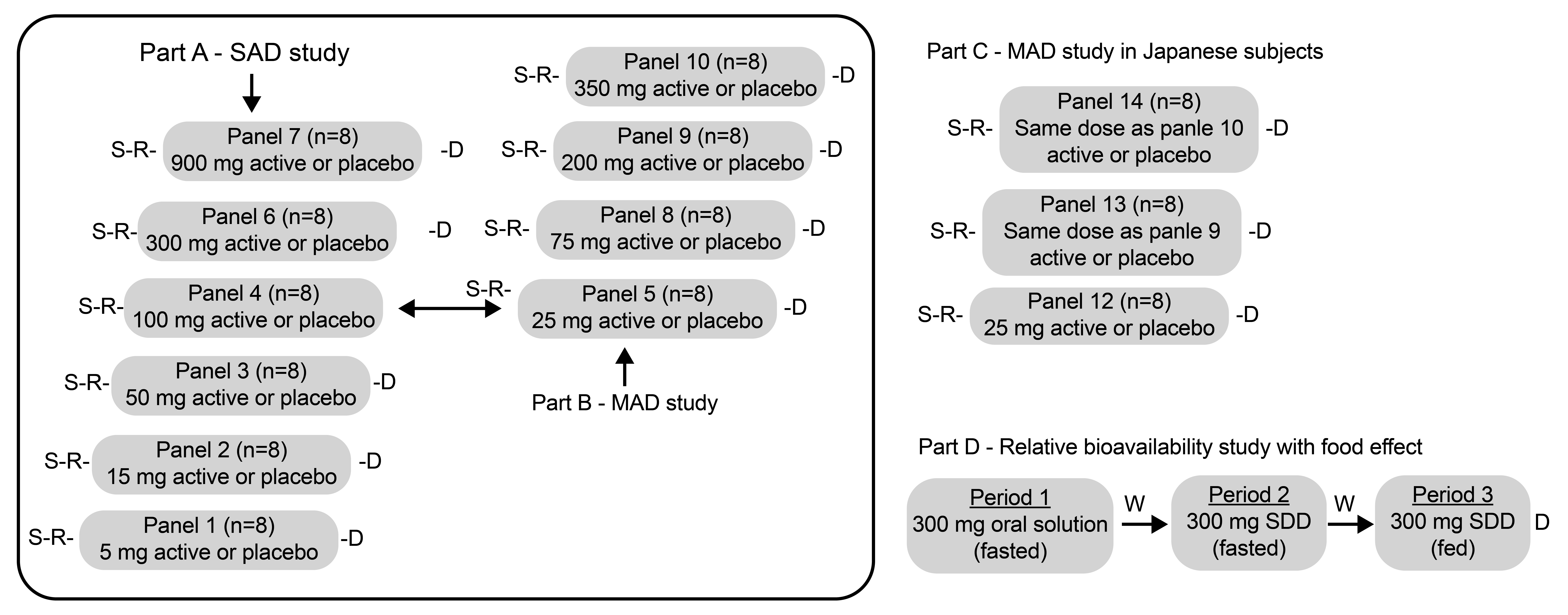

Supplement: Supplementary file 2 — High Resolution (TIFF 544 kb) [file 228_2017_2226_MOESM1_ESM.tif]

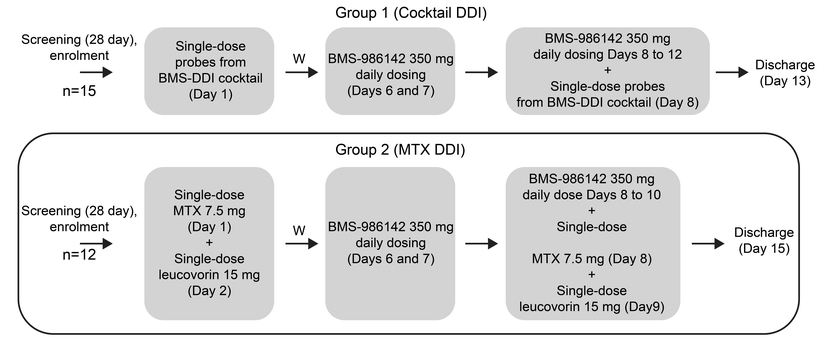

Supplement: Supplementary file 3 — Study 2 design. Demarcation represents methotrexate drug-drug interaction study (MTX DDI). DDI drug–drug interaction, MTX methotrexate, W washout (GIF 65 kb) [file 228_2017_2226_Fig6_ESM.gif]

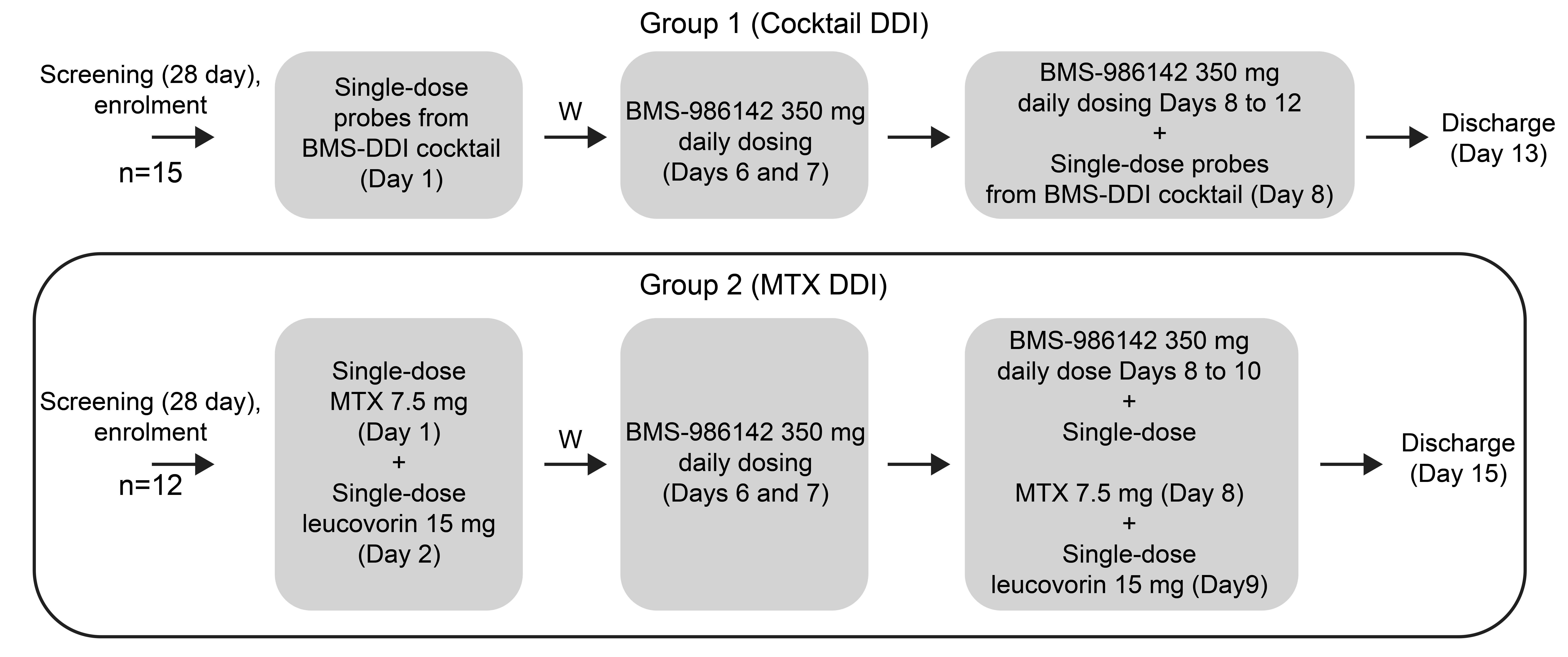

Supplement: Supplementary file 4 — High Resolution (TIFF 510 kb) [file 228_2017_2226_MOESM2_ESM.tif]

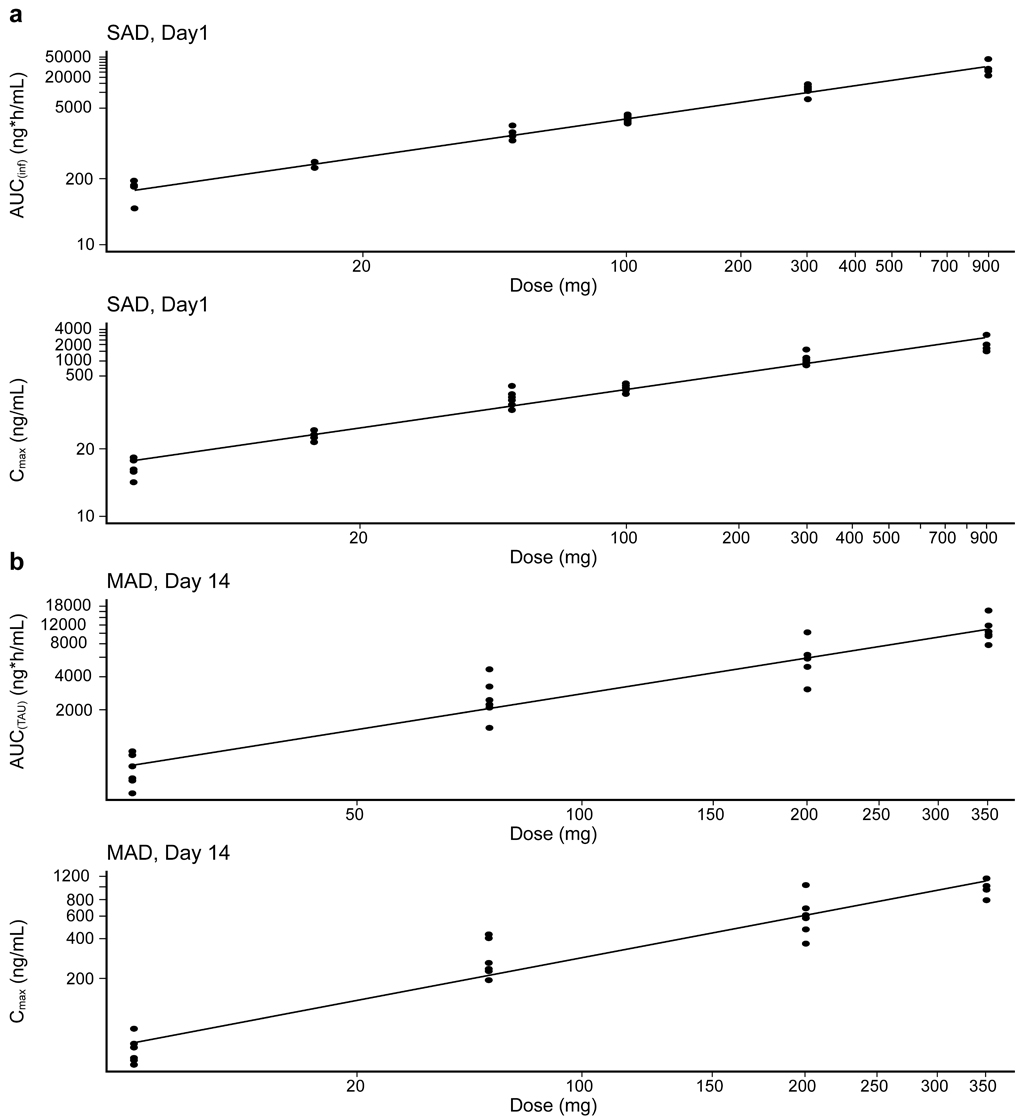

Supplement: Supplementary file 10 — Dose proportionality (fitted regression line) of Cmax and AUC with a) single ascending dose and b) multiple ascending dose administration (JPEG 141 kb) [file 228_2017_2226_Fig7_ESM.jpg]

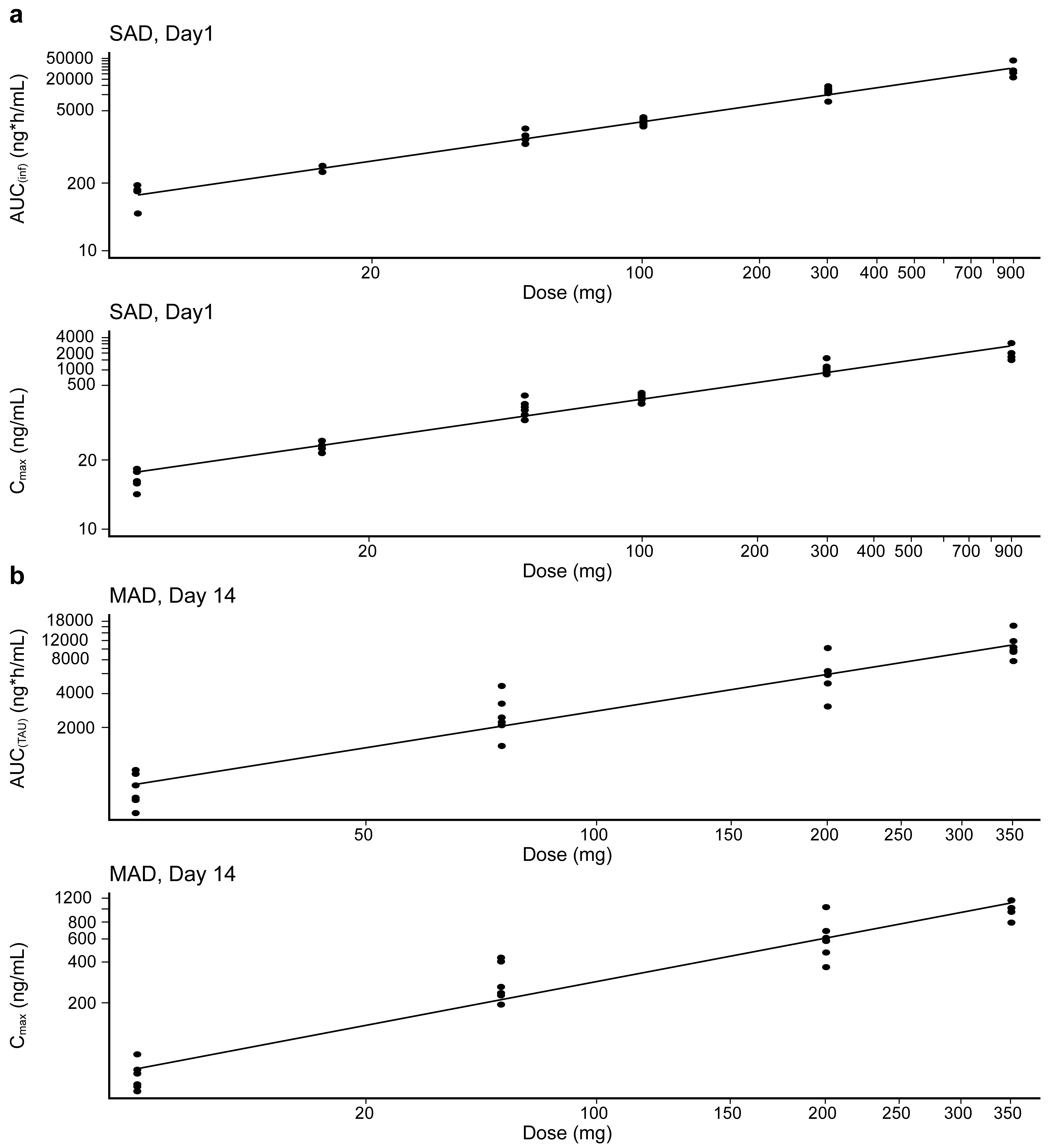

Supplement: Supplementary file 11 — High Resolution (TIFF 427 kb) [file 228_2017_2226_MOESM8_ESM.tif]
