# Supplementary material for: Safety, pharmacokinetics, and pharmacodynamics of BMS-986142, a novel reversible BTK inhibitor, in healthy participants
Source: Eur J Clin Pharmacol. 2017 Mar 6;73(6):689–98. doi: 10.1007/s00228-017-2226-2 (PMC5423977; doi:10.1007/s00228-017-2226-2)
Supplement: Supplementary file 5 — (DOCX 41 kb) [file 228_2017_2226_MOESM3_ESM.docx]

**Online Resource 3.** Patient demographic and baseline characteristics at screening (Study 1 and 2).

|  | Study 1 | | Study 2 |
| --- | --- | --- | --- |
| Characteristics | SAD  n = 48 | MAD  n = 32 | MTX + BMS-986142  n = 12 |
| Age |  |  |  |
| Mean (SD) | 38.8 (10.1) | 37.7 (9.2) | 33.6 (5.23) |
| Median | 37.5 | 37.5 | 33.0 |
| Min, Max | 20, 55 | 19, 52 | 25, 42 |
| Gender, n (%) |  |  |  |
| Male | 44 (91.7) | 31 (96.9) | 12 (100) |
| Female | 4 (8.3) | 1 (3.1) | 0 |
| Race, n(%) |  |  |  |
| White | 26 (54.2) | 19 (59.4) | 8 (66.7) |
| African American/Black | 15 (31.3) | 10 (31.3) | 4 (33.3) |
| Chinese | 2 (4.2) | 1 (3.1) | 0 |
| Japanese | 1 (2.1) | 0 | 0 |
| Asian other | 1 (2.1) | 0 | 0 |
| Native Hawaiian or other  Pacific Islander | 1 (2.1) | 0 | 0 |
| Other | 2 (4.2) | 2 (6.3) | 0 |
| Weight, kg |  |  |  |
| Mean (SD) | 82.6 (12.3) | 82.11 (11.5)^1^ | 82.78 (11.3) |
| Min, Max | 60.2, 110.9 | 59.7, 108.3 | 62.5, 107.8 |
| Body Mass Index, kg/m^2^ |  |  |  |
| Mean (SD) | 26.6 (2.7) | 26.0 (2.8)^1^ | 25.76 (2.6) |
| Min, Max | 19.8, 31.7 | 21.4, 30.7 | 20.5, 29.8 |

^1^n=31. *MAD* multiple ascending dose, *MTX* methotrexate, *SAD* single ascending dose, *SD* standard deviation.
