# Supplementary material for: Safety, pharmacokinetics, and pharmacodynamics of BMS-986142, a novel reversible BTK inhibitor, in healthy participants
Source: Eur J Clin Pharmacol. 2017 Mar 6;73(6):689–98. doi: 10.1007/s00228-017-2226-2 (PMC5423977; doi:10.1007/s00228-017-2226-2)
Supplement: Supplementary file 6 — (DOCX 50 kb) [file 228_2017_2226_MOESM4_ESM.docx]

**Online Resource 4:** Summary of adverse events for Study 1, a) SAD and b) MAD.

| a. SAD | | | | | | | | | |
| --- | --- | --- | --- | --- | --- | --- | --- | --- | --- |
| System organ class, n (%)  Preferred term, n (%) | Placebo  n = 12 | BMS-986142 5mg  n = 6 | BMS-986142 15mg  n = 6 | BMS-986142 50mg  n = 6 | BMS-986142 100mg  n = 6 | BMS-986142 300mg  n = 6 | BMS-986142 900mg  n = 6 | Any BMS-986142  n = 36 | Total  n = 48 |
| Total participants with an event | 2 (16.7) | 0 | 1 (16.7) | 2 (33.3) | 2 (33.3) | 1 (16.7) | 2 (33.3) | 8 (22.2) | 10 (20.8) |
| Gastrointestinal Disorders | 1 (8.3) | 0 | 1 (16.7) | 0 | 1 (16.7) | 1 (16.7) | 2 (33.3) | 5 (13.9) | 6 (12.5) |
| Aphthous stomatitis | 0 | 0 | 1 (16.7) | 0 | 0 | 0 | 0 | 1 (2.8) | 1 (2.1) |
| Diarrhoea | 0 | 0 | 0 | 0 | 0 | 0 | 1 (16.7) | 1 (2.8) | 1 (2.1) |
| Lip Dry | 0 | 0 | 0 | 0 | 0 | 1 (16.7) | 0 | 1 (2.8) | 1 (2.1) |
| Nausea | 0 | 0 | 0 | 0 | 0 | 0 | 1 (16.7) | 1 (2.8) | 1 (2.1) |
| Toothache | 1 (8.3) | 0 | 0 | 0 | 0 | 0 | 0 | 0 | 1 (2.1) |
| Vomiting | 0 | 0 | 0 | 0 | 1 (16.7) | 0 | 0 | 1 (2.8) | 1 (2.1) |
| Nervous system disorders | 1 (8.3) | 0 | 0 | 2 (33.3) | 1 (16.7) | 0 | 2 (33.3) | 5 (13.9) | 6 (12.5) |
| Headache | 1 (8.3) | 0 | 0 | 2 (33.3) | 0 | 0 | 2 (33.3) | 4 (11.1) | 5 (10.4) |
| Paraesthesia | 1 (8.3) | 0 | 0 | 0 | 0 | 0 | 1 (16.7) | 1 (2.8) | 2 (4.2) |
| Dizziness | 0 | 0 | 0 | 0 | 0 | 0 | 1 (16.7) | 1 (2.8) | 1 (2.1) |
| Syncope | 1 (8.3) | 0 | 0 | 0 | 0 | 0 | 0 | 0 | 1 (2.1) |
| Tension headache | 0 | 0 | 0 | 0 | 1 (16.7) | 0 | 0 | 1 (2.8) | 1 (2.1) |
| Infections and infestations | 0 | 0 | 0 | 1 (16.7) | 0 | 0 | 1 (16.7) | 2 (5.6) | 2 (4.2) |
| Upper respiratory tract  infection | 0 | 0 | 0 | 1 (16.7) | 0 | 0 | 1 (16.7) | 2 (5.6) | 2 (4.2) |
| General disorders and administration site conditions | 0 | 0 | 0 | 0 | 0 | 0 | 1 (16.7) | 1 (2.8) | 1 (2.1) |
| Chest pain | 0 | 0 | 0 | 0 | 0 | 0 | 1 (16.7) | 1 (2.8) | 1 (2.1) |
| Injury, poisoning and procedural complications | 0 | 0 | 0 | 1 (16.7) | 0 | 0 | 0 | 1 (2.8) | 1 (2.1) |
| Contusion | 0 | 0 | 0 | 1 (16.7) | 0 | 0 | 0 | 1 (2.8) | 1 (2.1) |
| Thermal burn | 0 | 0 | 0 | 1 (16.7) | 0 | 0 | 0 | 1 (2.8) | 1 (2.1) |
| Musculoskeletal and connective tissue disorders | 0 | 0 | 0 | 1 (16.7) | 0 | 0 | 0 | 1 (2.8) | 1 (2.1) |
| Muscle spasms | 0 | 0 | 0 | 1 (16.7) | 0 | 0 | 0 | 1 (2.8) | 1 (2.1) |
| Renal and urinary disorders | 0 | 0 | 0 | 0 | 1 (16.7) | 0 | 0 | 1 (2.8) | 1 (2.1) |
| Polyuria | 0 | 0 | 0 | 0 | 1 (16.7) | 0 | 0 | 1 (2.8) | 1 (2.1) |
| Respiratory, thoracic and mediastinal disorders | 0 | 0 | 0 | 0 | 0 | 0 | 1 (16.7) | 1 (2.8) | 1 (2.1) |
| Cough | 0 | 0 | 0 | 0 | 0 | 0 | 1 (16.7) | 1 (2.8) | 1 (2.1) |

| b. MAD |  |  |  |  |  |  |  |  |  |
| --- | --- | --- | --- | --- | --- | --- | --- | --- | --- |
| System organ class (%)  Preferred term (%) | Placebo  n = 8 | BMS-986142 25mg  n = 6 | BMS-986142 75mg  n = 6 | BMS-986142 200mg  n = 6 | BMS-986142 350mg  n = 6 | Any BMS-986142  n = 24 | Total  n = 32 |  |  |
| Total subjects with an event | 4 (50.0) | 2 (33.3) | 4 (66.7) | 1 (16.7) | 1 (16.7) | 8 (33.3) | 12 (37.5) |  |  |
| Nervous system disorders | 3 (37.5) | 0 | 1 (16.7) | 0 | 0 | 1 (4.2) | 4 (12.5) |  |  |
| Headache | 1 (12.5) | 0 | 1 (16.7) | 0 | 0 | 1 (4.2) | 2 (6.3) |  |  |
| Somnolence | 1 (12.5) | 0 | 0 | 0 | 0 | 0 | 1 (3.1) |  |  |
| Syncope | 1 (12.5) | 0 | 0 | 0 | 0 | 0 | 1 (3.1) |  |  |
| Respiratory, thoracic and mediastinal disorders | 1 (12.5) | 1 (16.7) | 1 (16.7) | 0 | 0 | 2 (8.3) | 3 (9.4) |  |  |
| Cough | 1 (12.5) | 1 (16.7) | 0 | 0 | 0 | 1 (4.2) | 2 (6.3) |  |  |
| Haemoptysis | 0 | 0 | 1 (16.7) | 0 | 0 | 1 (4.2) | 1 (3.1) |  |  |
| Throat irritation | 0 | 0 | 1 (16.7) | 0 | 0 | 1 (4.2) | 1 (3.1) |  |  |
| Gastrointestinal disorders | 0 | 0 | 2 (33.3) | 0 | 0 | 2 (8.3) | 2 (6.3) |  |  |
| Abdominal pain | 0 | 0 | 1 (16.7) | 0 | 0 | 1 (4.2) | 1 (3.1) |  |  |
| Diarrhoea | 0 | 0 | 1 (16.7) | 0 | 0 | 1 (4.2) | 1 (3.1) |  |  |
| Ear and labyrinth disorders | 0 | 0 | 1 (16.7) | 0 | 0 | 1 (4.2) | 1 (3.1) |  |  |
| Cerumen impaction | 0 | 0 | 1 (16.7) | 0 | 0 | 1 (4.2) | 1 (3.1) |  |  |
| Eye disorders | 0 | 1 (16.7) | 0 | 0 | 0 | 1 (4.2) | 1 (3.1) |  |  |
| Eye pain | 0 | 1 (16.7) | 0 | 0 | 0 | 1 (4.2) | 1 (3.1) |  |  |
| Infections and infestations | 0 | 0 | 0 | 1 (16.7) | 0 | 1 (4.2) | 1 (3.1) |  |  |
| Upper respiratory tract infection | 0 | 0 | 0 | 1 (16.7) | 0 | 1 (4.2) | 1 (3.1) |  |  |
| Injury, poisoning and procedural complications | 0 | 1 (16.7) | 0 | 0 | 0 | 1 (4.2) | 1 (3.1) |  |  |
| Thermal burn | 0 | 1 (16.7) | 0 | 0 | 0 | 1 (4.2) | 1 (3.1) |  |  |
| Investigations | 1 (12.5) | 0 | 0 | 0 | 0 | 0 | 1 (3.1) |  |  |
| Blood creatinine phosphokinase increased | 1 (12.5) | 0 | 0 | 0 | 0 | 0 | 1 (3.1) |  |  |
| Musculoskeletal and connective tissue disorders | 0 | 0 | 0 | 1 (16.7) | 0 | 1 (4.2) | 1 (3.1) |  |  |
| Back pain | 0 | 0 | 0 | 1 (16.7) | 0 | 1 (4.2) | 1 (3.1) |  |  |
| Psychiatric disorders | 0 | 0 | 1 (16.7) | 0 | 0 | 1 (4.2) | 1 (3.1) |  |  |
| Psychotic disorder | 0 | 0 | 1 (16.7) | 0 | 0 | 1 (4.2) | 1 (3.1) |  |  |
| Skin and subcutaneous tissue disorders | 0 | 0 | 0 | 0 | 1 (16.7) | 1 (4.2) | 1 (3.1) |  |  |
| Rash | 0 | 0 | 0 | 0 | 1 (16.7) | 1 (4.2) | 1 (3.1) |  |  |

*MAD* multiple ascending dose, *SAD* single ascending dose
