# Supplementary material for: Safety, pharmacokinetics, and pharmacodynamics of BMS-986142, a novel reversible BTK inhibitor, in healthy participants
Source: Eur J Clin Pharmacol. 2017 Mar 6;73(6):689–98. doi: 10.1007/s00228-017-2226-2 (PMC5423977; doi:10.1007/s00228-017-2226-2)
Supplement: Supplementary file 7 — (DOCX 40 kb) [file 228_2017_2226_MOESM5_ESM.docx]

**Online Resource 5**. Frequency distribution of maximum post-dose ∆QTcF in SAD.

|  |  | Maximum change from baseline in QTcF interval (msec) | | |
| --- | --- | --- | --- | --- |
| Treatment | n | ≤30 msec  N (%) | >30-≤60 msec  N (%) | >60 msec  N (%) |
| Placebo | 12 | 12 (100) | 0 | 0 |
| BMS-986142 5 mg | 6 | 6 (100) | 0 | 0 |
| BMS-986142 15 mg | 6 | 6 (100) | 0 | 0 |
| BMS-986142 50 mg | 6 | 6 (100) | 0 | 0 |
| BMS-986142 100 mg | 6 | 6 (100) | 0 | 0 |
| BMS-986142 300 mg | 6 | 6 (100) | 0 | 0 |
| BMS-986142 900 mg | 6 | 6 (100) | 0 | 0 |

*SAD* single ascending dose
