# Supplementary material for: Safety, pharmacokinetics, and pharmacodynamics of BMS-986142, a novel reversible BTK inhibitor, in healthy participants
Source: Eur J Clin Pharmacol. 2017 Mar 6;73(6):689–98. doi: 10.1007/s00228-017-2226-2 (PMC5423977; doi:10.1007/s00228-017-2226-2)
Supplement: Supplementary file 9 — (DOCX 41 kb) [file 228_2017_2226_MOESM7_ESM.docx]

| **Online Resource 7.** Summary of adverse events in Study 2 (MTX DDI). | | | | |
| --- | --- | --- | --- | --- |
| System Organ Class, n (%)  Preferred Term, n (%) | Single-dose MTX 7.5 mg (Day 1) + Single-dose Leucovorin 15 mg (Day 2)  n = 12 | BMS-986142 350 mg QD (Days 6 and 7)  n = 12 | BMS-986142 350 mg QD (Days 8 to 10) + Single-dose MTX 7.5 mg (Day 8) + Single-dose Leucovorin 15 mg (Day 9)  n = 12 | Total  n = 12 |
| Total Participants with an Event | 1 (8.3) | 2 (16.7) | 4 (33.3) | 4 (33.3) |
| Gastrointestinal Disorders | 0 | 0 | 2 (16.7) | 2 (16.7) |
| Nausea | 0 | 0 | 2 (16.7) | 2 (16.7) |
| Nervous System Disorders | 1 (8.3) | 1 (8.3) | 2 (16.7) | 2 (16.7) |
| Dizziness | 1 (8.3) | 0 | 2 (16.7) | 2 (16.7) |
| Headache | 0 | 1 (8.3) | 1 (8.3) | 1 (8.3) |
| Tremor | 1 (8.3) | 0 | 0 | 1 (8.3) |
| Psychiatric Disorders | 0 | 1 (8.3) | 1 (8.3) | 2 (16.7) |
| Anxiety | 0 | 0 | 1 (8.3) | 1 (8.3) |
| Sleep Disorder | 0 | 1 (8.3) | 0 | 1 (8.3) |
| General Disorders and Administration Site Conditions | 1 (8.3) | 0 | 1 (8.3) | 1 (8.3) |
| Asthenia | 1 (8.3) | 0 | 0 | 1 (8.3) |
| Fatigue | 0 | 0 | 1 (8.3) | 1 (8.3) |
| Infections and Infestations | 0 | 0 | 1 (8.3) | 1 (8.3) |
| Viral Infection | 0 | 0 | 1 (8.3) | 1 (8.3) |
| Injury, Poisoning, and Procedural Complications | 0 | 0 | 1 (8.3) | 1 (8.3) |
| Arthropod Bite | 0 | 0 | 1 (8.3) | 1 (8.3) |

*DDI* drug-drug interaction, *MTX* methotrexate
