# Supplementary material for: Safety, pharmacokinetics, and pharmacodynamics of BMS-986142, a novel reversible BTK inhibitor, in healthy participants
Source: Eur J Clin Pharmacol. 2017 Mar 6;73(6):689–98. doi: 10.1007/s00228-017-2226-2 (PMC5423977; doi:10.1007/s00228-017-2226-2)
Supplement: Supplementary file 12 — (DOCX 41 kb) [file 228_2017_2226_MOESM9_ESM.docx]

| **Online Resource 9.** Dose proportionality assessment for BMS-986142 PK parameters (evaluable PK population - SAD, Day 1 and MAD, steady state). | | | |
| --- | --- | --- | --- |
| Parameter | Unit | Estimated Slope | 90% CI of the Slope |
| SAD |  |  |  |
| C_max_ | ng/mL | 1.050 | (0.988, 1.112) |
| AUC_(0-T)_ | h*ng/mL | 1.125 | (1.071, 1.178) |
| AUC_(inf)_ | h*ng/mL | 1.089 | (1.036, 1.141) |
| MAD |  |  |  |
| C_max_ | ng/mL | 1.007 | (0.967, 1.186) |
| AUC_(TAU)_ | h*ng/mL | 1.104 | (0.977, 1.232) |
| *AUC_(0-T)_* area under the plasma concentration-time curve from time zero to the time of the last quantifiable concentration, *AUC_(inf)_* area under the plasma concentration-time curve from time zero extrapolated to infinite time, *AUC_(TAU)_* area under the plasma concentration-time curve in one dosing interval, *CI* confidence interval, *C_max_* maximum observed plasma concentration, *MAD* multiple ascending dose, *PK* pharmacokinetic, *SAD* single ascending dose. | | | |
