# Supplementary material for: Safety, pharmacokinetics, and pharmacodynamics of BMS-986142, a novel reversible BTK inhibitor, in healthy participants
Source: Eur J Clin Pharmacol. 2017 Mar 6;73(6):689–98. doi: 10.1007/s00228-017-2226-2 (PMC5423977; doi:10.1007/s00228-017-2226-2)
Supplement: Supplementary file 13 — Analytical methods for the determination of BMS-986142 or MTX in the human plasma (DOCX 50 kb) [file 228_2017_2226_MOESM10_ESM.docx]

**Online Resource 10.** Bioanalytical method.

1.1 Chemicals and reagents

Acetonitrile (HPLC grade) and ammonium bicarbonate were purchased from Sigma Aldrich (St. Louis, MO). formic acid (SupraPur), and ammonium hydroxide (ACS grade) were purchased from EMD Sciences (Gibbstown, NJ). HPLC grade methanol, 2-propanol, and dimethyl sulfoxide (DMSO) were purchased from JT Baker (Phillipsburg, NJ). Methyl-tert Butyl Ether (MTBE), was purchased from Honeywell Burdick and Jackson (Muskegon, MI). Deionized water was generated using a NANOpure® Diamond™ ultra pure water system from Barnstead International (Dubuque, IA). Control human K2EDTA plasma and human sodium heparin plasma were obtained from Bioreclamation, Inc. (Westbury, NY). BMS-986142 and the stable isotope labeled internal standard were synthesized at Bristol-Myers Squibb (Princeton, NJ). Methotrexate was purchased from USP (Rockville, MD) and 7-hydroxymethotrexate was purchased from Molcan Corporation (Toronto, Canada).

1.2 LC-MS/MS conditions

LC–MS/MS of BMS-986142 was carried out using a Shimadzu (Columbia, MD) Nexera HPLC system interfaced to a Sciex API 4000 (Foster City, CA) mass spectrometer equipped with a TurboIonsprayTM source. A Waters (Milford, MA) Acquity UPLC BEH C18 column (1.7 µm, 2.1 x 50 mm) was used for chromatographic separation and the mobile phases consisted of mobile phase A (MPA: 5 mM Ammonium Bicarbonate containing 0.005% Ammonium Hydroxide in 95% Water and 5% Acetonitrile) and mobile phase B (MPB: 5:95 water:acetonitrile) at a flow rate of 0.8 mL/min. The gradient started with 30% B held for 0.30 min and increased to 95% B in 1.30 min, then held at 95% B for 0.40 min, then switched back to 30% B in 0.05 min and held at 30% B until the run was stopped at 2.50 min. The injection volume was 5 μL. The column temperature was maintained at 50 °C and the autosampler temperature at 5 °C.

Positive ion electrospray mass spectrometry was used for the detection of BMS-986142 and its internal standard. The source temperature was set to 450°C. The ion spray voltage and entrance potential (EP) were maintained at 4500 and 10 V, respectively. UHP nitrogen (99.999%) at 30 and 6 psi were used as the curtain gas and collision gas, respectively. Analytes were detected using multiple reaction monitoring (MRM) and the optimized collision energy was 44 V for BMS-986142 and its internal standard. The transitions of m/z 573 → 538 and m/z 577 → 542 were monitored for BMS-986142 and the internal standard, respectively.

1.3 Sample preparation

Two stock solutions of metformin were prepared by dissolving the accurately weighed reference standards in DMSO/acetonitrile (50:50, v/v) to reach a ﬁnal concentration of 1 mg/mL. One stock solution was used to prepare standard samples in plasma, and the other stock solution was used to prepare quality control samples in plasma. A stock solution of internal standard was prepared at 0.2 mg/mL using DMSO/acetonitrile (50:50, v/v). All stock solutions were stored at -20°C. Standards and quality control (QC) samples were prepared using an initial spike of stock solution into K2EDTA human plasma blank, which was then diluted to obtain different concentration levels. Eight standards at the concentrations of 1–1000 ng/mL were used for the calibration curve. Double blanks (blank processed without internal standard) and single blanks (blank processed with internal standard) were prepared using the same matrix. Six levels of quality control samples were prepared for the evaluation of accuracy and precision. Standards and quality control samples were then pipetted into polypropylene tubes and stored at −20°C until analysis.

Samples were thawed at room temperature followed by mixing to ensure homogeneity. A liquid liquid extraction (LLE) method was used to extract the analyte from plasma samples. An aliquot of 50 µL plasma was transferred into a well of 96-deep-well plate and then mixed with 50 µL of internal standard working solution at 500 ng/mL in water/acetonitrile (50:50, v/v). After adding 50 µL of extraction buffer (1 M ammonium acetate), the plate was vortex for 10 seconds. Subsequently, 500 µL of LLE extraction solvent MTBE was added and mixed on linear shaker for 15 min. The mixture was then centrifuged for 10 min at 1522 × g, 4°C. 50 µL of supernatant was transferred to a new 96-deep-well plate and evaporated to dryness under nitrogen. After reconstituting using 150 µL of 70:30 MPA:MPB, an aliquot of 5 µL was injected onto LC–MS/MS system.

For the extraction of methotrexate and 7-hydroxymethotrexate, a 50 µL matrix aliquot was fortified with 50 µL of a 250 ng/mL internal standard working solution. Analytes were isolated through solid phase extraction using Waters Oasis MAX, 10 mg, 96-well SPE plates. After washing with 5% ammonium hydroxide followed by methanol, the analytes were eluted with 90:10:2 methanol/water/formic acid, v/v/v. The eluate was evaporated under a nitrogen stream at approximately 50°C, and the remaining residue was reconstituted with 250 µL of 15:85:0.5 acetonitrile/water/formic acid, v/v/v. The final extract was analyzed via HPLC with MS/MS detection.

1.4 Data processing and quantification

Data acquisition was performed using Analyst™ software. For each analytical run, peak area ratios of analyte and its internal standard were plotted versus the nominal concentrations of calibration standards using a linear least-squares regression with a weighting factor of 1/x2. The regression model and weighting function were selected based on a statistical analysis of data from accuracy and precision runs. Regression analysis and ANOVA analysis were performed in Watson LIMS. The regression equation for the calibration curve was used to back-calculate the measured concentrations for standards and QC samples, and the results were compared to the nominal concentrations to obtain the accuracy, expressed as a percent deviation (%) from the nominal value.

1.5 Method validation

Method validation was carried out according to the Bristol-Myers Squibb Standard Operating Procedures (SOPs) for chromatographic method validation which conforms to the U.S. FDA’s Good Laboratory Practice (GLP) regulations (21 CFR part 58). The validation experiments included but were not limited to the determination of the lower limit of quantification (LLOQ), specificity, accuracy and precision, recovery, and analyte stability under the expected conditions of sample transfer, sample storage, and sample preparation. The LLOQ of the assay was determined to be 1 ng/mL using six different lots of plasma. No interference from the endogenous components and the internal standard was observed.

The accuracy and precision data for BMS-986142, based on the maximum value from all six QC levels, obtained using one-way ANOVA in Watson are listed in Table 1. The inter-assay precision and accuracy for methotrexate and 7-hydroxymethotrexate are listed in Tables 2 and 3, respectively. The recovery of the analyte from human plasma during extraction was determined at 3 ng/mL and 800 ng/mL for BMS-986142, at 3 ng/mL, 30 ng/mL and 750 ng/mL for methotrexate and 7-hydroxymethotrexate, by comparing the response ratios in human plasma samples spiked with the analyte prior to extraction with those spiked post-extraction. The recovery for BMS-986142 is 89-90%. The recoveries for methotrexate and 7-hydroxymethotrexate are 88-94% and 83-88%, respectively. The room temperature, freeze-thaw, and frozen storage stability of BMS-986142 were evaluated in triplicate or quadruplicate using Low QC, High QC, and Dilution QC. The reinjection integrity was assessed by re-injecting an entire run. The deviations of the mean predicted concentrations of the test samples from the nominal concentrations were used as an indicator of the stability of the analyte. The stability data for BMS-986142 are summarized in Table 4. The stability data for methotrexate and 7-hydroxymethotrexate using Low QC and High QC are summarized in Tables 5 and 6, respectively.

Table 1. Accuracy and Precision for BMS-986142 in human EDTA plasma

| Nominal Conc.  (ng/mL) | LLOQ  (1.00) | Low  (3.00) | GM  (40.00) | Mid  (500.00) | High  (800.00) | Dilution  (10000.00) |
| --- | --- | --- | --- | --- | --- | --- |
| Mean Observed Conc. | 0.99 | 2.99 | 40.44 | 498.85 | 784.98 | 10037.60 |
| %Dev | -1.0 | -0.3 | 1.1 | -0.2 | -1.9 | 0.4 |
| Between Run Precision (%CV) | 6.1 | 4.6 | 3.8 | 4.6 | 5.6 | 6.8 |
| Within Run Precision (%CV) | 14.0 | 9.9 | 7.2 | 6.7 | 8.3 | 9.9 |
| Total Variation (%CV) | 15.2 | 10.9 | 8.2 | 8.1 | 10.0 | 12.0 |
| n | 21 | 28 | 28 | 28 | 28 | 24 |
| Number of Runs | 4 | 5 | 5 | 5 | 5 | 4 |

Table 2. Inter-assay accuracy and Precision for methotrexate in human sodium heparin plasma

| Nominal Conc.  (ng/mL) | QC0  (1.00) | QC1  (3.00) | QC2  (7.50) | QC3  (30.0) | QC5  (125) | QC6  (750) |
| --- | --- | --- | --- | --- | --- | --- |
| Mean Observed Conc. | 0.97 | 3.09 | 7.72 | 31.8 | 131 | 753 |
| S.D. | 0.09 | 0.14 | 0.33 | 1.70 | 5.12 | 43.0 |
| Between Run Precision (%CV) | 9.3 | 4.4 | 4.3 | 5.3 | 3.9 | 5.7 |
| %Dev | -3.0 | 3.0 | 3.0 | 5.9 | 5.1 | 0.3 |
| n | 18 | 18 | 18 | 18 | 18 | 18 |
| Number of Runs | 3 | 3 | 3 | 3 | 3 | 3 |

Table 3. Inter-assay accuracy and Precision for 7-hydroxymethotrexate in human sodium heparin plasma

| Nominal Conc.  (ng/mL) | QC0  (1.00) | QC1  (3.00) | QC2  (7.50) | QC3  (30.0) | QC5  (125) | QC6  (750) |
| --- | --- | --- | --- | --- | --- | --- |
| Mean Observed Conc. | 1.03 | 3.08 | 7.77 | 31.3 | 130 | 739 |
| S.D. | 0.15 | 0.21 | 0.31 | 2.01 | 7.79 | 44.9 |
| Between Run Precision (%CV) | 14.8 | 6.8 | 3.9 | 6.4 | 6.0 | 6.1 |
| %Dev | 2.6 | 2.7 | 3.6 | 4.3 | 4.0 | -1.4 |
| n | 18 | 18 | 18 | 18 | 18 | 18 |
| Number of Runs | 3 | 3 | 3 | 3 | 3 | 3 |

Table 4. Stability of BMS-986142

| QC Type  (Nominal Conc., ng/mL) | Low QC  3.00 | | High QC  800.00 | | Dilution QC  10000.00 | |
| --- | --- | --- | --- | --- | --- | --- |
| Sample Condition | Mean Conc. | %Dev | Mean Conc. | %Dev | Mean Conc. | %Dev |
| 19.6 hours at RT in Plasma | 3.25 | 8.3 | 816.24 | 2.0 | 10314.35 | 3.1 |
| After 4 Freeze-Thaw Cycle in Plasma | 2.81 | -6.3 | 752.66 | -5.9 | 9241.55 | -7.6 |
| 327 day at -20 °C in Plasma | 3.15 | 5.0 | 815.36 | 1.9 | 9578.40 | -4.2 |
| 7 days at 5 °C Re-injection integrity | 3.08 | 2.7 | 837.90 | 4.7 | 10986.50 | 9.9 |
| 96 h Processed sample stability | 3.12 | 4.0 | 813.52 | 1.7 | 9666.77 | -3.3 |

Table 5. Stability of methotrexate

| QC Type  (Nominal Conc., ng/mL) | Low QC  3.00 | | High QC  750 | |
| --- | --- | --- | --- | --- |
| Sample Condition | Mean Conc. | %Dev | Mean Conc. | %Dev |
| 24 hours at RT in Plasma | 3.00 | -0.1 | 751 | 0.2 |
| After 4 Freeze-Thaw Cycle in Plasma | 3.11 | 3.7 | 734 | -2.1 |
| 449 day at -20 °C in Plasma | 2.70 | -9.9 | 755 | 0.6 |
| 6 days at 5 °C Re-injection integrity | 3.10 | 3.4 | 734 | -2.2 |
| 142 h Processed sample stability | 2.72 | -9.2 | 652 | -13.1 |

Table 6. Stability of 7-hydroxymethotrexate

| QC Type  (Nominal Conc., ng/mL) | Low QC  3.00 | | High QC  750 | |
| --- | --- | --- | --- | --- |
| Sample Condition | Mean Conc. | %Dev | Mean Conc. | %Dev |
| 24 hours at RT in Plasma | 3.28 | 9.2 | 747 | -0.3 |
| After 4 Freeze-Thaw Cycle in Plasma | 3.04 | 1.4 | 751 | 0.2 |
| 449 day at -20 °C in Plasma | 2.69 | -10.4 | 747 | -0.3 |
| 6 days at 5 °C Re-injection integrity | 3.17 | 5.7 | 753 | 0.4 |
| 142 h Processed sample stability | 2.74 | -8.7 | 689 | -8.1 |
